# Chromosomal Microarray Analysis as First-Tier Genetic Test for Schizophrenia

**DOI:** 10.3389/fgene.2021.620496

**Published:** 2021-10-01

**Authors:** Chia-Hsiang Chen, Min-Chih Cheng, Tsung-Ming Hu, Lieh-Yung Ping

**Affiliations:** ^1^ Department of Psychiatry, Chang Gung Memorial Hospital, Taoyuan, Taiwan; ^2^ Department and Graduate Institute of Biomedical Sciences, Chang Gung University, Taoyuan, Taiwan; ^3^ Department of Psychiatry, Yuli Branch, Taipei Veterans General Hospital, Hualien, Taiwan

**Keywords:** copy number variation, schizophrenia, chromosome microarray analysis, genetic test, rare mutation

## Abstract

Schizophrenia is a chronic, devastating mental disorder with complex genetic components. Given the advancements in the molecular genetic research of schizophrenia in recent years, there is still a lack of genetic tests that can be used in clinical settings. Chromosomal microarray analysis (CMA) has been used as first-tier genetic testing for congenital abnormalities, developmental delay, and autism spectrum disorders. This study attempted to gain some experience in applying chromosomal microarray analysis as a first-tier genetic test for patients with schizophrenia. We consecutively enrolled patients with schizophrenia spectrum disorder from a clinical setting and conducted genome-wide copy number variation (CNV) analysis using a chromosomal microarray platform. We followed the 2020 “Technical Standards for the interpretation and reporting of constitutional copy-number variants: a joint consensus recommendation of the American College of Medical Genetics and Genomics (ACMG) and the Clinical Genome Resource (ClinGen)” to interpret the clinical significance of CNVs detected from patients. We recruited a total of 60 patients (36 females and 24 males) into this study. We detected three pathogenic CNVs and one likely pathogenic CNV in four patients, respectively. The detection rate was 6.7% (4/60, 95% CI: 0.004–0.13), comparable with previous studies in the literature. Also, we detected thirteen CNVs classified as uncertain clinical significance in nine patients. Detecting these CNVs can help establish the molecular genetic diagnosis of schizophrenia patients and provide helpful information for genetic counseling and clinical management. Also, it can increase our understanding of the pathogenesis of schizophrenia. Hence, we suggest CMA is a valuable genetic tool and considered first-tier genetic testing for schizophrenia spectrum disorders in clinical settings.

## Introduction

Schizophrenia is a chronic debilitating mental disorder marked by delusions, hallucinations, erratic emotions, bizarre behaviors, and cognitive deficits. Approximately 1% of the general population is affected by the disease. It is a complex disorder involving genetic and environmental factors, and genetic factors play a significant role in the genesis of schizophrenia. Recent advances in genetic and genomic technology have significantly increased our understanding of the genetic architecture of schizophrenia (Avramopoulos, 2018). Genetic variants associated with schizophrenia are heterogeneous, including hundreds of common variants with modest clinical effects and multiple rare mutations with high penetrance and varied clinical expressivity (Foley et al., 2017; Henriksen et al., 2017).

Copy number variations (CNVs) are deleted and duplicated genomic DNA segments resulting from aberrant chromosomal rearrangement. They are associated with human health and diseases (Harel and Lupski, 2018; Hu et al., 2018). CNVs are divided into common and rare CNVs according to their populational frequencies. Accumulating evidence indicates that rare CNVs contribute significantly to the genetic basis of neuropsychiatric disorders, such as intellectual disability, autism spectrums disorder (ASD), and schizophrenia (Rutkowski et al., 2017; Lowther et al., 2017a). Identifying disease-associated CNVs can help establish cell and animal models of neuropsychiatric disorders to elucidate the pathogenesis and facilitate new therapeutic regimens (Nomura and Takumi, 2012; Flaherty and Brennand, 2017; Rutkowski et al., 2017; Takumi and Tamada, 2018).

The Psychiatric Genomics Consortium CNV Analysis Group performed a genome-wide CNV analysis in 21,094 patients with schizophrenia and 20,227 controls. They confirmed a global enrichment of CNV burden in patients with schizophrenia compared to controls. Additionally, they reported rare recurrent CNVs at eight genetic loci reaching a genome-wide significance. The odds ratios of CNVs at these eight loci range from 3.8 to infinity (Marshall et al., 2017), supporting a substantial role of rare CNVs in the genesis of schizophrenia.

Chromosomal microarray analysis (CMA) is a molecular genetic tool that can identify the location and the size of CNV with high resolution and precision (Rosenfeld and Patel, 2017; Shaikh, 2017). It has been recommended as the first-tier genetic test for patients with developmental disabilities or congenital abnormalities, as it significantly increases the diagnostic yield of children affected with global developmental delay, autism spectrum disorders, or multiple congenital abnormalities (Manning et al., 2010; Miller et al., 2010; Michelson et al., 2011). Costain and colleagues conducted a prospective community-based chromosomal microarray analysis of 459 patients with schizophrenia in a catchment area. They found that 8.1% of their study subjects carried rare large (>500 kb) CNVs of clinical significance. Hence, they suggested using CMA as a genetic test for schizophrenia (Costain et al., 2013). Lowther and colleagues reported a CNV survey of 546 patients with schizophrenia from six community clinics; they found 39/546 (7.1%) patients had at least one pathogenic CNV (Lowther et al., 2017b). Baker and colleagues also proposed CMA as a routine clinical genetic test for schizophrenia (Baker et al., 2014), in view that identification of pathogenic CNVs can help establish the genetic diagnosis and improve the medical and mental health care of patients with schizophrenia.

We previously reported three novel rare CNVs associated with schizophrenia in three families, respectively (Liao et al., 2012), indicating the potential clinical utility of chromosomal microarray as a genetic test for schizophrenia in our population. This study attempted to gain some experiences of applying CMA as a first-tier genetic test for schizophrenia spectrum disorders in a clinical setting. We consecutively recruited patients with schizophrenia in our clinical practice and conducted a genome-wide CNV analysis for them. Here, we report our findings in a sample of 60 patients recruited from the outpatient unit of our hospital.

## Materials and Methods

### Subjects

All the patients were residents of Taiwan. We recruited patients fulfilling the diagnostic criteria of schizophrenia (295.9) or schizoaffective disorder (295.70) according to the DSM-5 (Diagnostic and Statistical Manual of Mental Disorder-5th edition) into this study. After interviewing and reviewing their medical records, patients with a history of substance abuse, intellectual disability, childhood-onset psychiatric disorders, head injury, or medical conditions associated with psychiatric disorders were excluded. The Review Board of Chang Gung Memorial Hospital-Linkou approved the study with the approved number of 104-6522A3 (201506522A3). We obtained informed consent from each participant after a full explanation of this study.

### Genome-Wide Copy Number Variation Analysis

We extracted genomic DNA from venous blood from each participant using the Smart Genomic DNA Extraction kit (Intelligent Biomedicine, Taipei, Taiwan) according to the manufacturer’s instructions. DNA was subjected to genome-wide copy number variation analysis using CytoScan HD Array (Affymetrix Inc., Santa Clara, CA, United States) following the manufacturer’s protocol. The Genomic Medicine Core Laboratory of Chang Gung Memorial Hospital-Linkou (Taoyuan, Taiwan) did the experiment, and the raw data were analyzed using the software Chromosomal Analysis Suite Version 3.3.0.139 (r10838) (Affymetrix Inc., Santa Clara, CA, United States). Gain and loss of CNVs were analyzed at the resolution of 50 probes and 100 kb. The genomic coordinates of CNVs followed the human genome sequences version GRCh37/hg19.

### Real-Time Quantitative PCR

The real-time quantitative PCR (RT-qPCR) method was used as a complementary method to verify the authenticity of CNVs detected by the CytoScan HD array. Genes encompassed by the CNV of interest were selected. A comparative ddCt method was used for the analysis of RT-qPCR data. The Ct of the target gene was subtracted by the Ct of a reference gene (dCt), then normalized to a control subject (ddCt). The relative fold change to a normal subject was determined as 2^−ddCt^. We performed experiments using the StepOnePlus machine (Applied Biosystems, Forster City, CA) with the SYBR green method according to the manufacturer’s instructions, the experiments. Target genes, primer sequences, optimal annealing temperature, and the amplicon sizes are listed in Supplementary Table S1.

### Assessment of the Pathogenicity of Copy Number Variation

To assess the clinical significance of CNVs detected from patients, we followed the “Technical Standards for the interpretation and reporting of constitutional copy-number variants: a joint consensus recommendation of the American College of Medical Genetics and Genomics (ACMG) and the Clinical Genome Resource (ClinGen)” (Riggs et al., 2020). This report recommended using a semiquantitative, point-based scoring system to classify CNVs into five categories used in sequence variation classification (Richards et al., 2015), including pathogenic, likely pathogenic, uncertain significance, likely benign, and benign. We used the recommended web-based ClinGen CNV Pathogenicity Calculator (http://cnvcalc.clinicalgenome.org/cnvcalc/) to record and calculate the final score of CNVs detected in our sample. CNVs with a score of equal or greater than 0.99 were classified pathogenic, while scores between 0.90 and 0.98 were likely pathogenic. CNVs with scores between −0.89 and 0.89 belonged to the uncertain significance (VUS) category, while scores between −0.90 and −0.98 belonged to the likely benign category. CNVs with scores less than −0.99 were classified as benign. We defined pathogenic and likely pathogenic CNVs as of clinical relevance in this study.

## Results

### Identification of Pathogenic and Likely Pathogenic Copy Number Variations

**FIGURE 1:**
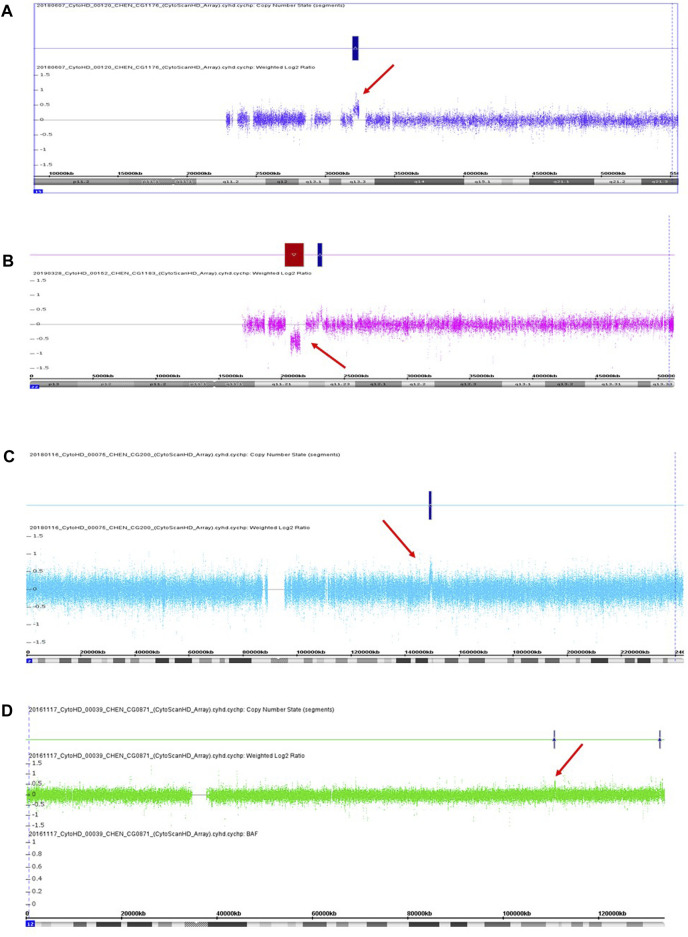
Three pathogenic CNVs **(A–C)** and one likely pathogenic CNV **(D)** were detected in this study. **(A)** A 433 kb microduplication at 15q13.3 in the patient CG1176. **(B)** A 1,489 kb microdeletion at 22q11.21 in the patient CG1183. **(C)** A 912 kb microduplication at 2q13.1 in the patient CG0200. **(D)** A 188 kb microduplication at 12q24.11 in the patient CG0871. Red arrow indicates the location of CNV.

We recruited 60 patients into this study from May 2016 to April 2019, including 36 females and 24 males. The age ranged from 20 to 70 years old. The mean age was 41 years old, with a standard deviation of 12 years old. We detected three pathogenic CNVs and one likely pathogenic CNV in four respective patients from this sample. The three pathogenic CNVs included a 443 kb microduplication at 15q13.3, a 1,489 kb microdeletion at 22q11.21, and a 912 kb microduplication at 2q23.1. The likely pathogenic CNV was a 188 kb microduplication at 12q24.11. The readouts of three pathogenic CNVs and one likely pathogenic CNV in the Chromosomal Analysis Suite are shown in Figure 1. The authenticity of these CNVs was further verified using real-time quantitative PCR. The detection rate of pathogenic and likely pathogenic CNVs together in this sample was 6.7% (4/60, 95% CI: 0.004–0.13). The genetic information of these four clinically relevant CNVs, including their location, size, and encompassed genes, is listed in Table 1.

**TABLE 1:** Pathogenic and likely pathogenic CNVs detected in this study.

Patient	Sex	Location	Start (bp)	End (bp)	Size (kb)	Type	Genes	Genes	Category
CG1176	M	15q13.3	32,003,537	32,446,830	443	Dup	2	*OTUD7A* *^a^*(X)*, CHRNA7* *^a^*(X)	Pathogenic
CG1183	F	22q11.21	20,311,903	21,800,797	1,489	Del	36	*LOC101927859, FAM230A, TMEM191B, PI4KAP1, RIMBP3, LINC01660, ZNF74, SCARF2* *^a^* *, KLHL22, MED15, POM121L4P, TMEM191A, PI4KA* *^a^* *, SERPIND1, SNAP29, CRKL, LINC01637, AIFM3, LZTR1* *^a^* *, THAP7, THAP7-AS1, TUBA3FP, P2RX6, SLC7A4, MIR649, P2RX6P, LRRC74B, BCRP2, LOC102724728, FAM230B, GGT2, POM121L8P, LOC100996335, RIMBP3C, RIMBP3B, HIC2*	Pathogenic
CG0200	F	2q23.1	149,065,069	149,976,617	912	Dup	4	*MBD5* *^a^*(X)*, EPC2, KIF5C* *^a^* *, LYPD6B*(X)	Pathogenic
CG0871	F	12q24.11	110,748,734	110,937,025	188	Dup	6	*ATP2A2* *^a^*(X)*, ANAPC7, ARPC3, GPN3, FAM216A, VPS29* *^a^*(X)	Likely pathogenic

a Indicates verified by real-time quantitative PCR; Dup indicates duplication, Del indicates deletion. (X): indicates gene disrupted by CNV breakpoint.

The first pathogenic CNV was a 433 kb microduplication at 15q13.3, which was detected in a male patient (CG1176) without a family history of mental illness. He suffered from several acute psychotic episodes in his twenties and was subsequently diagnosed with schizophrenia. His psychotic symptoms included auditory hallucination, the idea of reference, a delusion of persecution, irritability, and agitation. He had an unremarkable birth history and normal psychosocial development before the onset of his mental illness. Also, he did not have physical abnormalities, an intellectual disability, or neurological symptoms. His psychotic symptoms were under control after antipsychotics treatment, but he still suffered from residual auditory hallucinations. Currently, he keeps a job and has good compliance with long-term antipsychotic medication.

The second pathogenic CNV was a 1,489 kb microdeletion at 22q11.21, present in a female patient without a family history. Her birth history and psychosocial development were unremarkable before the onset of her mental illness. She did not have physical abnormalities, an intellectual disability, or neurological symptoms. In her twenties, she started to express psychotic symptoms of loosening of association, irrelevant speech, unstable emotions, and auditory hallucinations and was subsequently diagnosed with schizoaffective disorder. She received antipsychotics and mood stabilizer treatment and had a good response, but she did not comply with medication well due to the lack of insight. Her mental symptoms recurred several times after discontinuation of her medication, which led to her social function impairment.

The third pathogenic CNV was a 912 kb microduplication at 2q23.1, occurring in a female patient without a family history of mental illness. In her twenties, she was diagnosed with schizophrenia due to psychotic symptoms, such as auditory hallucinations, the idea of reference, delusions of being controlled and possessed. She did not have physical abnormalities, an intellectual disability, or neurological symptoms. Also, her birth history and normal psychosocial development were unremarkable before the onset of her mental illness. She attempted suicide once due to commanding hallucinations. Her social function deteriorated despite continuous antipsychotic treatment.

The likely pathogenic CNV was a 188 kb microduplication at 12q24.11, identified in a female patient without a family history of mental illness. She was diagnosed with schizoaffective disorder in her twenties. Her mental symptoms included auditory hallucination, loose association, and irrelevant speech. Also, she had depressive episodes in her course of illness. She had normal psychosocial development before the onset of her mental illness. Also, she did not have physical abnormalities, an intellectual disability, or neurological symptoms. She had a reasonable response to antipsychotic and antidepressant treatment.

### Identification of Copy Number Variations With Uncertain Significance

Furthermore, we detected thirteen CNVs classified as uncertain significance in nine patients. The authenticity of these thirteen CNVs was also verified using real-time quantitative PCR. The genetic information of these thirteen CNVs with uncertain significance, including their location, size, and encompassed genes, is listed in Table 2. The patient CG0200 carried one CNV with uncertain significance (a microduplication at 4q13.1) and one pathogenic CNV (a microduplication at 2q23.1). The patient CG0027 carried two CNVs with uncertain significance, while the patient CG0314 carried four CNVs with uncertain significance. The other six patients carried only one CNV with uncertain significance.

**TABLE 2:** CNVs with uncertain significance detected in this study.

Patient	Sex	Location	Start	End	Size (kb)	Type	Genes	Genes
CG0027	F	Xp11.23	49,025,922	49,133,609	108	Dup	9	*PLP2, PRICKLE3, SYP* *^a^* *, SYP-AS1, CACNA1F, CCDC22* *^a^* *, FOXP3, FLICR, PPP1R3F*
		Xq28	152,720,486	152,845,900	125	Dup	3	*HAUS7, BGN, ATP2B3* *^a^*
CG0314	F	7q36.2	153,524,141	153,648,708	125	Dup	1	*DPP6* *^a^*
		7q36.3	158,392,720	158,798,052	405	Dup	3	*NCAPG2* *^a^* *, ESYT2, WDR60*
		8q22.3	104,459,758	104,586,655	127	Dup	2	*LOC105375690, RIMS2* *^a^*
		Xq28	152,740,638	152,845,920	105	Dup	3	*HAUS7, BGN, ATP2B3* *^a^*
CG0888	M	16q23.3	83,996,531	84,454,763	458	Dup	12	*OSGIN1, NECAB2* *^a^* *, SLC38A8, MBTPS1, HSDL1, DNAAF1, TAF1C, ADAD2, LOC654780, KCNG4, WFDC1, ATP2C2* *^a^*
CG0903	M	22q11.22	22,332,040	22,578,983	247	Dup	2	*TOP3B* *^a^* *, PRAMENP*
CG0029	M	16p13.3	1,052,879	1,268,271	216	Dup	4	*SSTR5-AS1, SSTR5* *^a^* *, C1QTNF8, CACNA1H* *^a^*
CG0200	F	4q13.1	62,632,588	62,779,822	147	Dup	1	*LPHN3* *^a^*
CG0368	F	1p32.3	53,329,117	53,744,865	416	Dup	13	*ZYG11A, ECHDC2, SCP2, MIR1273F, MIR5095, MIR1273G, PODN, SLC1A7, CPT2, C1orf123, MAGOH, LOC100507564, LRP8* *^a^*
CG0310	F	18p11.31	4,403,535	4,930,841	527	Dup	1	*DLGAP1* *^a^*
CG0663	F	20p12.1	14,602,445	14,895,154	293	Del	3	*MACROD2* *^a^* *, MACROD2-IT1, MACROD2-AS1*

a Indicates verified by real-time quantitative PCR; Dup indicates duplication, Del indicates deletion.

## Discussion

CMA has been used as a first-tier genetic test in patients with intellectual disability, congenital abnormalities, and autism spectrum disorders (Miller et al., 2010), and several studies suggested the clinical utility of CMA for schizophrenia and other psychiatric disorders (Costain et al., 2013; Baker et al., 2014; Lowther et al., 2017a; Lowther et al., 2017b). We conducted CMA in a sample of patients with schizophrenia or schizoaffective recruited consecutively from a clinical setting to test the utility of CMA in searching for the genetic etiology for schizophrenia patients. We selected patients without comorbidity of other diagnoses, such as substance abuse, head injury, medical conditions associated with brain dysfunction, and neurological disorders. We also excluded patients with the comorbid diagnosis with intellectual disability, as one study reported that schizophrenia with a low intelligence quotient (IQ) had a higher pathogenic CNV rate than those without low IQ (Lowther et al., 2017b).

We detected four clinically relevant CNVs in four respective patients. The detection rate was 6.7%, comparable to the prevalence rate of 8.1% in a community-based sample of 595 unrelated patients (Costain et al., 2013), and the 7.1% in a study of 546 community-based patients (Lowther et al., 2017b). Together, these studies support that CMA is an instrumental tool in detecting the genetic etiology in a portion of schizophrenia patients. Identifying clinically relevant CNVs in schizophrenia can provide helpful information for genetic counseling and guide clinical management for patients in clinical settings. (Baker et al., 2014). Furthermore, the identification of clinically relevant CNVs in schizophrenia can increase our understanding of the pathogenesis of schizophrenia.

The first pathogenic CN = V reported in this study was a 443 kb microduplication at 15q13.3 in the male patient CG1176. The breakpoints of the microduplication intersected with the *OTUD7A* and *CHRNA7*, respectively, resulting in the disruption of these two genes. CNVs at 15q13.3, including microdeletion and microduplication, are associated with multiple neuropsychiatric disorders, such as epilepsy, intellectual disability, autism spectrum disorders, and schizophrenia (Gillentine and Schaaf, 2015). There are seven genes at the 15q13.3 CNV region, including *FAN1, MTMR10, TRPM1, MIR211, KLF13, OTUD7A*, and *CHRNA7*. Several lines of study have suggested that *CHRNA7* is a candidate gene for the neuropsychiatric phenotypes in patients with 15q13.3 CNVs (Gillentine and Schaaf, 2015; Gillentine et al., 2017). *CHRNA7* encodes the subunit protein to form the alpha-7 nicotinic receptor, a homopentameric ligand-gated ion channel regulating inhibitory and excitatory neurotransmission in the brain. Alpha-7 nicotinic receptor is involved in the molecular mechanism of attention, learning, and memory (Bouzat et al., 2018; Natarajan et al., 2020). Postmortem studies showed reduced immunoreactivity of alpha-7 nicotinic receptors in the brains of schizophrenia (Freedman et al., 2000). Hence, the alpha-7 nicotinic receptor’s reduced function was related to the sensory gating deficits and impaired cognitive function in schizophrenia. Alpha-7 nicotinic receptor has been considered the drug target for cognitive enhancement in schizophrenia patients (Freedman, 2014). Hence, disruption of the *CHRNA7* by the 443 kb microduplication may contribute to the schizophrenia pathogenesis of our patient.

The *OTUD7A* encodes a deubiquitinating enzyme highly expressed in the brain. Uddin and colleagues combined whole-genome sequencing, human brain gene expression analysis, and study of a mouse model with a syntenic heterozygous deletion of 15q13; they found reduced expression of *OTUD7A* in the dendritic spine of the cortical neurons that contributed to the dendrite outgrowth abnormalities in the heterozygous 15q13 deletion mice. They proposed that the *OTUD7A* was the major regulatory gene for 15q13.3 microdeletion syndrome phenotypes (Uddin et al., 2018). Similarly, Yin and colleagues studied Otud7a-knockout mice; they also found that Otud7a-null mice had decreased dendritic spine density and recapitulated many clinical features of 15q13.3 syndrome. They suggested that decreased *OTUD7A* dosage was the major contributor to the neurodevelopmental phenotypes associated with 15q13.3 microdeletion syndrome due to the aberrant dendritic spine density and activity (Yin et al., 2018).

Further, mutations of the *OTUD7A* were associated with neurodevelopment disorders. Garret and colleagues reported a homozygous missense mutation of *OTUD7A* in a male patient with severe global developmental delay, language impairment, and epileptic encephalopathy. His parents and a younger brother who manifested learning disability were heterozygous carriers of the missense mutation (Garret et al., 2020). Suzuki and colleagues also reported the biallelic loss of function of *OTUD7A* in a male patient with hypotonia, intellectual disability, and seizures (Suzuki et al., 2021). These findings suggest the *OTUD7A* plays a critical role in brain development, and haploinsufficiency of the *OTUD7A* may contribute to the pathogenesis of our patient.

The second pathogenic CNV identified in this study was a 1,489 kb microdeletion at 22q11.21 in the female patient CG1183. 22q11.2 is a hot region with recurrent CNVs in humans associated with various neurodevelopmental disorders such as intellectual disability, autism spectrum disorders, and schizophrenia. Researchers classified CNVs at 22q11.2 as proximal, central, and distal according to their locations (Burnside, 2015; Woodward et al., 2019). The 1,489 kb microdeletion in this patient belonged to the central 22q11.21 deletion, encompassing 36 genes (Table 1). *SNAP29* is one of the genes deleted in our patient. SNAP29 is one of the SNARE (Soluble NSF Attachment REceptor) proteins that regulate a wide range of cellular functions. Studies showed that SNAP29 was involved in autophagy, synaptic transmission, cell division, and infection (Mastrodonato et al., 2018). A polymorphism at the promoter of the *SNAP29* was reported to be associated with schizophrenia (Saito et al., 2001; Wonodi et al., 2005). Using a functional genomic approach, Forsyth and colleagues discovered that genes in the 22q11.2 locus formed an extensive protein network and regulated the developmental modules associated with schizophrenia and autism spectrum disorders. They also suggested that *SEPT5*, *PI4KA*, and *SNAP29* were candidate drivers of 22q11.2 synaptic pathology relevant to schizophrenia and autism spectrum disorder (Forsyth et al., 2020). Both *PI4KA* and *SNAP29* were located at the deleted region in our patient. The 22q11.2 deletion syndrome working groups of the Enhancing Neuroimaging Genetics through Meta-Analysis (ENIGMA) recently reported that patients with 22q11.2 deletion syndrome had reduced cortical surface area and increased cortical thickness. They found that *AIFM3* was associated with the cortical surface area deviance, while *P2RX6*, was associated with cortical thickness deviance (Forsyth et al., 2021). These two genes were also included in the deleted region in our patient. Thus, the microdeletion in this patient contains several genes that may work together and leads to the pathogenesis of her clinical phenotypes.

The third pathogenic CNV detected in this study was a 912 kb microduplication at 2q23.1 in the female patient CG200. Four genes were present in this region, including two duplicated genes, *EPC2* and *KIF5C,* and two breakpoints-disrupted genes, *MBD5* and *LYPD6B*. *KIF5C* is a member of kinesin superfamily proteins that are molecular motors involved in cargo transport in the central nervous system (Duquesne et al., 2020). Pathogenic mutations *KIF5C* are rare but cause abnormal cortical development and result in severe developmental delay, absent language, seizures, and abnormal behaviors in affected patients (Michels et al., 2017; Duquesne et al., 2020), indicating the critical role of *KIF5C* in the normal development of the cortex. The duplication of *KIF5C* in our patient may lead to increased expression of KIF5C in the brain and contribute to the pathogenesis of schizophrenia, which is distinct from the clinical symptoms caused by loss-of-function *KIF5C* pathogenic variants.

The other important gene that may contribute to the pathogenesis of schizophrenia in this patient is the *MBD5. MBD5* encodes a member of the methyl-CpG-binding domain (MBD) family proteins which bind specifically to methylated DNA and regulate gene expression. Haploinsufficiency of *MBD5* causes neurodevelopmental disorders such as intellectual disability, autism spectrum disorders, and seizures. Furthermore, patients with haploinsufficiency of *MBD5* may present with psychiatric disorders, including anxiety, bipolar disorder, and schizophrenia (Hodge et al., 2014; Kushima et al., 2017), suggesting the variable expressivity of *MBD5* haploinsufficiency. The cause of *MBD5* haploinsufficiency can be due to 2q23.1 deletions encompassing the *MBD5*, intragenic deletion of *MBD5*, and pathogenic mutations of *MBD5* (Mullegama et al., 1993). The breakpoint of the 912 kb microduplication in the patient CG0200 intersected with the *MBD5*, leading to disruption of the *MBD5*. Our patient presented schizophrenia symptoms only. She did not present other *MBD5*-related neurodevelopmental symptoms such as developmental delay, speech impairment, seizure, and abnormal behaviors (Mullegama et al., 2015; Mullegama and Elsea, 2016), suggesting other factors may modify the clinical presentations of the *MBD5*-haploinsufficiency.

This study detected a 188 kb microduplication at 12q24.11, classified as likely pathogenic CNV in the female patient CG0871. This microduplication encompassed six genes, including *ATP2A2, ANAPC7, ARPC3, GPN3, FAM216A,* and *VPS29.* Among these genes, the *ATP2A2* and *VPS29* were interrupted by the breakpoints of the microduplication, respectively. *ATP2A2* encodes an ATPase, which helps pump the calcium ion from cytosol to sarcoplasmic reticulum lumen. Haploinsufficient mutations of the *ATP2A2* cause a rare autosomal dominant form of skin disorder, Darier disease (Ruiz-Perez et al., 1999). Patients with Darier disease have an increased risk for bipolar disorder and schizophrenia (Cederlöf et al., 2015; Gordon-Smith et al., 2018), indicating that mutations of *ATP2A2* have pleiotropic effects on the brain. Notably, our patient manifested schizophrenia symptoms only. She did not present any skin disorder, suggesting psychiatric manifestations might be independent of skin pathology in patients with *ATP2A2* haploinsufficiency. A large-scale genome-wide association study discovered that *ATP2A2* was significantly associated with schizophrenia, indicating that the *ATP2A2* was a risk gene for schizophrenia (Schizophrenia Working Group of the Psychiatric Genomics Consortium, 2014).

Another gene disrupted by the microduplication in this patient was the *VPS29* which encodes a subunit protein of the retromer complex. Retromer complex is involved in the recycling of proteins from endosomes to the plasma membrane. Dysfunction of the retromer complex is associated with some neurodegenerative disorders (Reitz, 2018). A recent study reported that a *VPS29* variant associated with decreased VPS29 expression in the brain significantly increased the risk for schizophrenia, suggesting reduced VPS29 expression in the postmortem brains of schizophrenia is a biological mechanism underlying schizophrenia (Byrne et al., 2020). Hence, the disruption of the *VPS29* in our patient might contribute to the pathogenesis of her schizophrenia. Further study is needed to address this issue.

In this patient, four genes, ANAPC7, ARPC3, GPN3, and FAM216A, were duplicated in the microduplication. In these four genes, *ARPC3* might implicate the schizophrenia pathogenesis of this patient. *ARPC3* encodes one of the seven subunits of the Arp2/3 protein complex, which is involved in the actin polymerization in cells (Welch et al., 1997). Several studies showed that disruption of *ARPC3* was associated with aberrant synaptic plasticity in the brain and behavioral abnormalities in mice (Kim et al., 2013; Spence et al., 2016; Kim et al., 2020). Nevertheless, whether overexpression of the *ARPC3* is associated with brain pathology and behavioral problems remains to be elucidated.

Besides four clinically relevant CNVs, we detected thirteen CNVs classified as uncertain significance in nine patients in this study. The patient CG0200 carried a microduplication of 147 kb at 4p13.1, classified as uncertain significance (Table 2), and a pathogenic microdeletion at 2q23.1 (Table 1). The microduplication at 4p13.1 intersected with the *LPHN3.* Variants of *LPHN3* were associated with attention deficit hyperactivity disorder, autism spectrum disorder, and substance use disorder (Martinez et al., 2016; Arcos-Burgos et al., 2019; Kappel et al., 2019). Hence, this CNV might contribute to the patient’s symptoms.

The female patient CG0027 carried two CNVs with uncertain significance, including a 108 kb microduplication at Xp11.23 and a 125 kb microduplication at Xq28 (Table 2). The 108 kb microduplication at Xp11,23 encompassed nine genes (Table 2). Among these genes, *PLP*2, *CCDC22*, and *SYP* mutations were associated with X-linked intellectual disability (Zhang et al., 2007; Tarpey et al., 2009; Kolanczyk et al., 2015). Some schizophrenia patients were found to have rare *SYP* mutations (Shen et al., 2012). Furthermore, the 125 kb microduplication at Xq28 encompassed the *ATP2B3* associated with X-linked spinocerebellar ataxia (Zanni et al., 2012; Feyma et al., 2016). This patient did not have an intellectual disability and other neurological symptoms. Hence, this patient’s psychiatric manifestations might be related to the random inactivation of these genes at the X chromosome and varied clinical symptoms.

The patient CG0314 carried four CNVs classified as uncertain significance. They were a 125 kb microduplication at 7q36.2, a 405 kb microduplication at 7q36.3, a 127 kb microduplication at 8q22.3, and a 105 kb microduplication at Xq28 (Table 2). The 105 kb microduplication at Xq28 also included the *ATP2B3,* whose mutations were associated with X-linked spinocerebellar ataxia (Zanni et al., 2012; Feyma et al., 2016). The 125 kb microduplication at 7q36.2 disrupted the *DPP6*, whose mutations were associated with autism (Marshall et al., 2008; Egger et al., 2014), autosomal dominant microcephaly, intellectual disability (Liao et al., 2013), and neurodegenerative dementia (Cacace et al., 2019). The 405 kb microduplication at 7q36.3 covered the *NCAGP2*, whose mutations were associated with microcephaly and intellectual disability (Perche et al., 2013; Khan et al., 2019). Finally, the 127 kb microduplication at 8q22.3 contained the *RIMS2,* which was proposed as a risk gene for autism (Fan et al., 2018a). Taken together, these four CNVs might work independently or synergistically to contribute to schizophrenia’s pathogenesis. Several studies reported that multiple CNVs might interact and result in varied clinical phenotypes of developmental and neuropsychiatric disorders, including intellectual disability, congenital abnormalities (Girirajan et al., 2012), autism spectrum disorder (Gau et al., 2012), and schizophrenia (Williams et al., 2013; Rudd et al., 2014; Kushima et al., 2017). Our findings of multiple CNVs in three patients in this study agreed with these studies and supported the multiple-hits hypothesis of schizophrenia. Notably, additional hits are not limited to CNV. They may include rare or common genetic variants with various effect sizes and non-genetic factors (Williams et al., 2013; Feigenson et al., 2014; Rudd et al., 2014).

Six patients (CG0888, CG0903, CG0029, CG0368, CG0310, and CG0663) carried one CNV with uncertain significance, respectively (Table 2). The 458 kb microduplication at 16q23.3 in the patient CG0888 encompassed the *NECAB2,* which encodes a neuronal calcium-binding protein that interacts with and modulates adenosine A2A receptor (Canela et al., 2007) and metabotropic glutamate receptor type 5 (Canela et al., 2009). The 247 kb microduplication at 22q11.22 in the patient CG0903 interrupted the *TOP3B*, whose mutations were associated with several neurodevelopmental disorders including schizophrenia, autism, and epilepsy (Stoll et al., 2013; Ahmad et al., 2017; Daghsni et al., 2018). The 216 kb microduplication at 16p13.3 in the patient CG0029 spanned the *SSTR5*. CNVs encompassing the *SSTR5* were reported in patients with schizophrenia before (Rodríguez-Santiago et al., 2010; Saus et al., 2010). The 416 kb microduplication at 1p32.3 in the patient CG0368 intersected with the *LRP8,* which encodes the low-density lipoprotein receptor-related protein 8. The *LRP8* was considered a susceptibility gene for schizophrenia and bipolar disorder (Li et al., 2016; Xiao et al., 2020). The 527 kb microduplication at 18p11.3 in the patient CG0310 interrupted the *DLGAP1,* which encodes the DLG associated protein 1. Rare missense mutations of the *DLGAP1* were detected in patients with schizophrenia before (Li et al., 2013; Xing et al., 2016), while common polymorphisms of the *DLGAP1* influenced the executive function in attention deficit hyperactivity disorder (Fan et al., 2018b). Finally, the 293 kb microdeletion at 20p12.1 in the patient CG0663 covered the *MACROD2* gene. CNVs involving the *MACROD2* gene were detected in patients with attention deficit hyperactivity disorder, autism (Lionel et al., 2011; Bacchelli et al., 2020), and major depression with suicide attempts (Perlis et al., 2012). Although these CNVs were classified as uncertain significance according to the guidelines used in this study, their relationship with schizophrenia needs further evaluation as more studies come along in the future.

In this study, we feedback our CNV fidnings to the patients and their families. We discussed the clinical significance of their CNV findings and answered their questions regarding the origin of their CNV findings and the possible treatment in the future, given the limited treatment based on the CNV findings at this moment. We also attempted to conduct a family study to trace the origins of three pathogenic CNVs and one likely pathogenic CNV identified in this study. However, we could not collect DNA from both parents because some parents passed away, divorced, separated, or were reluctant to donate their DNA. Hence, we were not able to report the origins of these four CNVs in this study.

In conclusion, given a small sample size of sixty patients recruited consecutively from a clinical setting, we identified three pathogenic CNVs, one likely pathogenic, and thirteen CNVs with uncertain significance in this study. Further, we found three patients carrying multiple CNVs. The detection of these CNVs enriches our understanding of the underlying genetic deficits associated with schizophrenia spectrum disorders. Hence, we suggest that CMA is a valuable tool for a first-tier genetic test for schizophrenia in clinical settings. It can help establish the patients’ molecular genetic diagnosis, provides helpful information for genetic counseling, and increases our understanding of the pathogenesis of schizophrenia.

## Data Availability

The original contributions presented in the study are included in the article/Supplementary Material, further inquiries can be directed to the corresponding author.
