# Supplementary material for: Chromosomal Microarray Analysis as First-Tier Genetic Test for Schizophrenia
Source: Front Genet. 2021 Oct 1;12:620496. doi: 10.3389/fgene.2021.620496 (PMC8517076; doi:10.3389/fgene.2021.620496)
Supplement: Supplementary file 1 [file Table1.DOCX]

**Supplementary Table 1**. Sequences of primers, annealing temperature, and size of amplicon for verification of CNVs detected in this study using real-time quantitative PCR.

|  | Forward | Reverse | Ta | Size (bp) |
| --- | --- | --- | --- | --- |
| OTUD7A Exon1 | 5’-AAGCGAGAGAAAATGGAAGTCG-3’ | 5’-ACGGAGCTCGGGAAGGAGACGG -3’ | 63 ^o^C | 190 |
| CHRNA7 Exon2 | 5’-TCTCTCCTTAAGTGTCCCTGCAA-3’ | 5’-CACCACGTCCATGATCTGCAGGA-3’ | 63 ^o^C | 155 |
| SCARF2 Exon10 | 5’-AACACACTCAACTGCAGCTTCCT-3’ | 5’-CACAGGGCCTTCATCAGTGGTGT-3’ | 63 ^o^C | 114 |
| PI4KA Exon6 | 5’-ATCCCTCCTCATTCCCTCCGTGT-3’ | 5’-CCCACCTGAGAGATGCTGGACAC-3’ | 63 ^o^C | 161 |
| PI4KA Exon24 | 5’-GGATTCTCTCTCTGTTGCTGCACCT-3’ | 5’-CCTCCAGCAGACAGACACGCATT-3’ | 63 ^o^C | 163 |
| LZTR1 Exon10 | 5’-TTCAGGTTGGTGGGGCTGAAGTG-3’ | 5’-GGATCCCAGCCTGTACCTCCGA-3’ | 63 ^o^C | 177 |
| ATP2A2 Exon 16 | 5’-TGACAGCAGCCCTTGGATTTCC-3’ | 5’-CCACCCGCTGATCAATGGTTCCT-3’ | 63 ^o^C | 167 |
| VPS29 Exon4 | 5’-GAGATCTGCACATCCCACACCGG-3’ | 5’-TGAACATCACCAGCCAGAGTCTTGA-3’ | 63 ^o^C | 151 |
| MBD5 Exon6 | 5’-TCGGGTACAAAGAGAGGCATCGA-3’ | 5’-CCCCTGGAGAAGTTGCTGCTGTT-3’ | 63 ^o^C | 191 |
| KIF5C Exon2 | 5’-GCTACCTCCCAACACGACCCAAG-3’ | 5’-AGGCACATTGATTCCTAGGCTGCA-3’ | 63 ^o^C | 150 |
| SYP Exon4 | 5’-CAGTGCTGTCTTCCTCACCCCTG-3’ | 5’-GTGTTGAGTCCCGAGGTCACAGG-3’ | 63 ^o^C | 218 |
| CCDC22 Exon2 | 5’-GCGATCAAGCTGGTCCCCTTCTT-3’ | 5’-TGTGTGCATTAGGAGGAGGGCC-3’ | 63 ^o^C | 232 |
| ATP2B3 Exon19 | 5’-TGAGTGCTGAAAATCCTGGCCGG-3’ | 5’-GGAGGTGGAGTGTGAAGCATGCT-3’ | 63 ^o^C | 163 |
| DPP6 Exon1 | 5’-GGCTTCGCTGTACCAGAGGTT-3’ | 5’-TACGTCCTCCTCGTCACCATC-3’ | 63 ^o^C | 244 |
| NCAPG2 Exon16 | 5’-AGTAAACGATGTTGCATGCATGGC-3’ | 5’-GCACAGAGGCAAACTTGTTAATCGTG-3’ | 63 ^o^C | 128 |
| RIMS2 Exon1 | 5’-CAGCCCGAGATGCCTGACCTCA-3’ | 5’-GGGCAGGGAGAGAGGCGGGAATAT-3’ | 63 ^o^C | 150 |
| NECAB2 Exon6 | 5’ –CACCTCCAATTTCCCTCCCACCC-3’ | 5’-GCTGGCTGGTCTGTTCCTCGATG-3’ | 63 ^o^C | 191 |
| ATP2C2 Exon3 | 5’-CACACTGGGCTGTCGGAGTTCTC-3’ | 5’-TTGAAAGATGGAATGGCTGCCCC-3’ | 63 ^o^C | 183 |
| TOP3B Exon1 | 5’-GAGCCACGGAACCTAAGAACGCC-3’ | 5’-GATCCAGCTCCGGTCCTTGTTCC-3’ | 63 ^o^C | 142 |
| SSTR5 Exon1 | 5’-CCTGGGTCCTGTCTCTGTGCATG-3’ | 5’-CACAGGCAGATGACCAGCAGC-3’ | 63 ^o^C | 172 |
| CACNA1H Exon4 | 5’-TGAGCTGTTCCACGGGCCCT-3’ | 5’-ATCCAGCCTGTTCCACGTGTCAC-3’ | 63 ^o^C | 154 |
| ADGRL3 Exon9 | 5’-TCGGACCACAACTTTGAGCCCAG-3’ | 5’-AGAGCTGGGATTTGGGACGATGC-3’ | 63 ^o^C | 150 |
| LRP8 Exon18 | 5’-GGAGACCAGAGAACCGGAAGA-3’ | 5’-GTTCATGCCCTCACTCACCAG-3’ | 63 ^o^C | 181 |
| DLGAP1 Exon1 | 5’-CCCCACCGTATTAACATGCACCCA-3 | 5’-CTATGCTGCCGATTCCCCGAGG-3’ | 63 ^o^C | 175 |
| MACROD2 Exon5 | 5’-TCTCTTCCTCTTCCCATCTTGAGCT-3 | 5’-TGCAGGAAGGTCATAGCCACATGT-3’ | 63 ^o^C | 192 |
